# Antimitochondrial Antibody-Negative Primary Biliary Cholangitis: A Diagnostic Challenge in a Sri Lankan Patient

**DOI:** 10.7759/cureus.105425

**Published:** 2026-03-18

**Authors:** Chathura Madhushan Angulugaha Angulugaha Gamage, Madhupawani Wijayasuriya Wijayasuriya Arachchige, Vajira Samarawickrama

**Affiliations:** 1 Department of Medicine, National Hospital of Sri Lanka, Colombo, LKA; 2 Department of Pathology and Laboratory Medicine, National Hospital of Sri Lanka, Colombo, LKA; 3 Department of Gastroenterology and Hepatology, National Hospital of Sri Lanka, Colombo, LKA

**Keywords:** ama-negative primary biliary cholangitis, cholestasis, liver biopsy, primary biliary cholangitis (pbc), pruritus

## Abstract

Primary biliary cholangitis (PBC) is a chronic autoimmune cholestatic liver disease characterized by progressive destruction of small intrahepatic bile ducts, in which antimitochondrial antibodies (AMA) are detected in the majority of patients and serve as a key diagnostic marker. A small subset of patients, however, remains AMA-negative, creating diagnostic uncertainty and potentially delaying treatment. We report the case of a 65-year-old woman who presented with progressive pruritus and fatigue and was found to have a predominantly cholestatic liver enzyme pattern. Extensive evaluation excluded extrahepatic biliary obstruction, viral hepatitis, and drug-induced liver injury. Despite a negative AMA test, a liver biopsy demonstrated portal inflammatory infiltrates with interface hepatitis and a prominent bile ductular reaction, establishing the diagnosis of AMA-negative PBC. The patient was commenced on ursodeoxycholic acid with partial symptomatic improvement. This case highlights the importance of maintaining clinical suspicion for PBC despite negative AMA and underscores the diagnostic value of liver biopsy in seronegative cases.

## Introduction

Primary biliary cholangitis (PBC) is an autoimmune cholestatic liver disease characterized by progressive destruction of the small intrahepatic bile ducts [1]. Antimitochondrial antibodies (AMA) are detected in the majority of patients and are considered the immunological hallmark of the disease [1,2]. According to major international guidelines such as those from the European Association for the Study of the Liver (EASL), the diagnosis of PBC can be established when at least two of the following are present: cholestatic liver enzyme abnormalities, positive AMA, and compatible histological findings in the absence of biliary obstruction. However, a small subset of patients are AMA-negative, which may lead to diagnostic uncertainty and delay. In such cases, diagnosis depends on compatible clinical features, exclusion of alternative causes of cholestasis, and supportive histopathological findings. Early recognition is important because untreated disease may progress to fibrosis and cirrhosis, while timely initiation of ursodeoxycholic acid can significantly improve long-term outcomes. Reports of AMA-negative PBC from Sri Lanka remain scarce. This case highlights the diagnostic challenges encountered in resource-limited settings where advanced serological panels may not be readily available.

## Case presentation

A 65-year-old woman with a 10-year history of diabetes mellitus and a six-year history of hypertension presented with a four-month history of generalized pruritus associated with nausea and intermittent vomiting. She also reported progressive fatigue over six months but denied constitutional symptoms such as weight loss or anorexia. There was no history of jaundice, alcohol use, or exposure to hepatotoxic medications.

Examination revealed no stigmata of chronic liver disease apart from excoriation marks. Liver function tests show elevated total bilirubin, alkaline phosphatase (ALP), and gamma-glutamyl transferase (gamma-GT) levels, with marginally elevated aspartate aminotransferase (AST) and alanine transaminase (ALT) levels. Autoimmune testing showed ANA (antinuclear antibody) positivity (1:100 nucleoplasm pattern) with repeatedly negative AMA, negative anti-smooth muscle antibodies, and anti-dsDNA antibodies. Additional PBC-specific autoantibodies, such as anti-gp210 and anti-sp100, were not available for testing in our setting. Serum immunoglobulin levels, including immunoglobulin M (IgM), were within the normal range. Viral hepatitis screening was negative, and thyroid-stimulating hormone levels were within the normal range. Magnetic resonance cholangiopancreatography (MRCP) revealed a normal intra- or extrahepatic biliary tree. Contrast-enhanced CT and ultrasound of the liver did not reveal any hepatic parenchymal changes or any extrahepatic duct dilatation (Table 1).

**Table 1:** Summary of investigations. MRCP: magnetic resonance cholangiopancreatography

Investigation	Result	Normal Range
White blood cells	11.4×10³/mm³	4.5–10.3×10³/mm³
Hemoglobin	12.1 g/dL	12.0–16.0 g/dL
Platelet	210×10^3^/mm³	150–450×10^3^/mm³
C-reactive protein	4 mg/L	< 6 mg/L
Aspartate aminotransferase	46 U/L	10–40 U/L
Alanine transaminase	78 U/L	7–56 U/L
Alkaline phosphatase	536 U/L	44–147 U/L
Gamma-glutamyl transferase	1600 U/L	5–48 U/L
Total bilirubin	3.1 mg/dL	0.2–1.3 mg/dL
Antinuclear antibody	Positive (1:100)	<1:80
Antimitochondrial antibody	Negative (repeated)	-
Anti-smooth muscle antibodies	Negative	-
MRCP	Normal biliary tree	-
Liver biopsy	Interface hepatitis with bridging fibrosis	-

**Figure 1:**
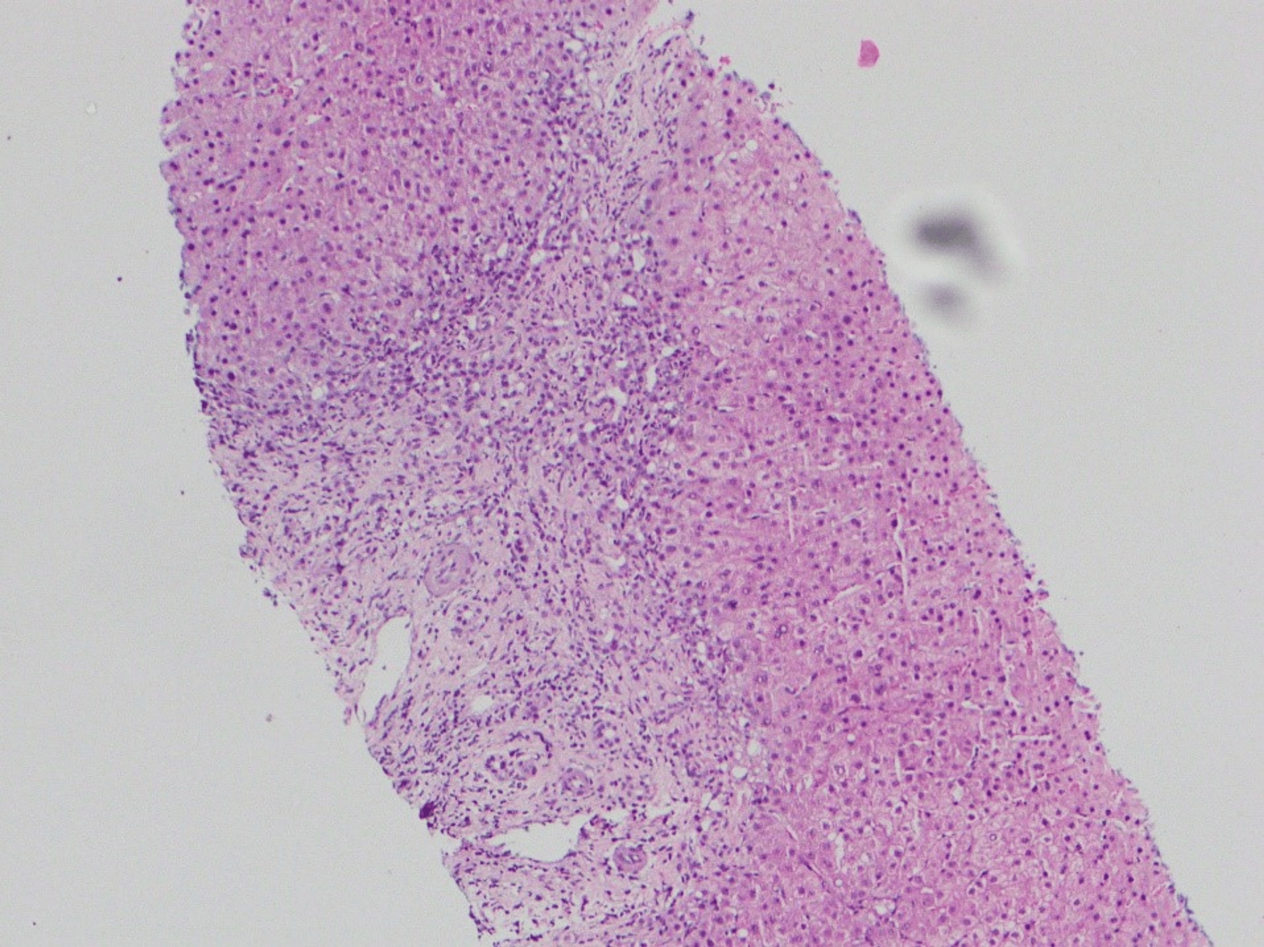
Liver biopsy showing portal-based inflammation (H&E stain, low power). Low-power photomicrograph demonstrating preserved hepatic architecture with portal tract expansion by inflammatory infiltrate and interface activity. H&E: hematoxylin and eosin

**Figure 2:**
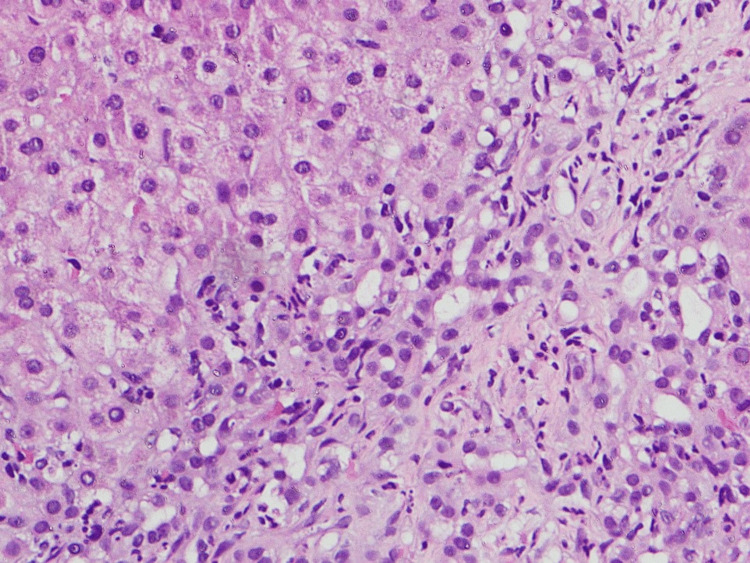
Liver biopsy showing interface hepatitis and hepatocyte injury (H&E stain, ×400). High-power view demonstrating portal inflammation with interface hepatitis, bile duct injury, and hepatocellular feathery degeneration consistent with cholestatic injury. No granulomas or significant steatosis are identified. These findings support the diagnosis of primary biliary cholangitis. H&E: hematoxylin and eosin

Given persistent cholestasis, a liver biopsy was performed. Histological examination of a single liver core containing seven portal tracts demonstrated moderate portal tract inflammation with bridging fibrosis and an inflammatory infiltrate predominantly composed of lymphocytes and neutrophils. There was associated moderate lobular inflammation with interface hepatitis. Many hepatocytes showed feathery degeneration. A prominent bile ductular reaction was also observed within the portal tracts. The vascular structures appeared unremarkable. No granuloma formation, bile plugs, significant plasma cell predominance, steatosis, diastase-resistant globules, or features of malignancy were identified (Figures 1, 2).

In the context of persistent cholestatic liver enzyme elevation, exclusion of extrahepatic obstruction, and compatible histology, a diagnosis of AMA-negative PBC was established. The patient was commenced on ursodeoxycholic acid 300 mg twice daily (600 mg/day) and cholestyramine. At the six-month follow-up, the patient reported improvement in pruritus. Liver function tests demonstrated only partial biochemical improvement: total bilirubin was 2.5 mg/dL, ALP remained elevated at 430 U/L, and transaminases were mildly elevated (AST 46 U/L, ALT 66 U/L), suggesting an incomplete biochemical response to ursodeoxycholic acid.

## Discussion

Current clinical practice guidelines from the EASL state that the diagnosis of PBC can be established when at least two of the following are present: cholestatic liver enzyme elevation, positive AMA, and compatible histology in the absence of biliary obstruction [3]. AMA-negative PBC represents a small but clinically important subgroup and may lead to diagnostic delay [4]. Reports describing AMA-negative PBC remain relatively limited, and the absence of the characteristic serological marker may increase the risk of missed or delayed diagnosis, particularly in resource-limited settings.

In the present case, two of the three diagnostic criteria were fulfilled, namely, persistent cholestatic liver enzyme elevation and histological features compatible with PBC, thereby supporting the diagnosis despite negative AMA. Although elevated IgM levels are commonly observed in PBC, normal IgM levels may occur and do not exclude the diagnosis. This highlights the importance of maintaining a high index of suspicion in patients with unexplained cholestasis.

Liver biopsy plays a crucial role in establishing the diagnosis in seronegative cases [5]. The presence of interface hepatitis and ANA positivity may raise the possibility of PBC-autoimmune hepatitis overlap syndrome. However, the patient did not fulfill the established diagnostic criteria for overlap syndrome, as transaminase elevations were modest, immunoglobulin levels were within the normal range, and anti-smooth muscle antibodies were negative. Therefore, the overall clinical and histological findings were considered more consistent with PBC.

The absence of significant biochemical improvement at six months may represent an incomplete biochemical response to ursodeoxycholic acid, which is recognized in a subset of patients with PBC [6]. In clinical practice, inadequate response to ursodeoxycholic acid is typically assessed after 12 months of therapy, with persistent elevation of ALP being an important indicator. Continued monitoring is therefore required to determine long-term treatment response. Although the principles of management are similar to those of classical PBC, variability in biochemical response may pose ongoing challenges in the long-term management of AMA-negative cases. Consideration of second-line therapy such as obeticholic acid or fibrates may be warranted if an inadequate response persists [7-10].

## Conclusions

AMA-negative PBC remains a diagnostic challenge and requires a high index of clinical suspicion in patients presenting with unexplained cholestasis. In seronegative cases, diagnosis relies on the integration of clinical features, cholestatic liver biochemistry, and compatible histological findings after exclusion of other causes of biliary obstruction. This case underscores the importance of considering PBC even in the absence of AMA and highlights the diagnostic value of liver biopsy. Further research and improved access to advanced serological markers may help facilitate earlier recognition of AMA-negative PBC. Early diagnosis and appropriate management are essential to prevent disease progression and optimize long-term outcomes.
